# Multiple perspectives on clinical decision support: a qualitative study of fifteen clinical and vendor organizations

**DOI:** 10.1186/s12911-015-0156-4

**Published:** 2015-04-24

**Authors:** Joan S Ash, Dean F Sittig, Carmit K McMullen, Adam Wright, Arwen Bunce, Vishnu Mohan, Deborah J Cohen, Blackford Middleton

**Affiliations:** Oregon Health & Science University, Portland, OR USA; University of Texas School of Biomedical Informatics, Houston, TX USA; Kaiser Permanente Center for Health Research, Portland, OR USA; Brigham and Women’s Hospital, Boston, MA USA; Harvard Medical School, Boston, MA USA; Partners HealthCare, Boston, MA USA; Vanderbilt University, Nashville, TN USA

**Keywords:** Clinical decision support, Knowledge management, Governance, Rapid assessment process

## Abstract

**Background:**

Computerized clinical decision support (CDS) can help hospitals to improve healthcare. However, CDS can be problematic. The purpose of this study was to discover how the views of clinical stakeholders, CDS content vendors, and EHR vendors are alike or different with respect to challenges in the development, management, and use of CDS.

**Methods:**

We conducted ethnographic fieldwork using a Rapid Assessment Process within ten clinical and five health information technology (HIT) vendor organizations. Using an inductive analytical approach, we generated themes from the clinical, content vendor, and electronic health record vendor perspectives and compared them.

**Results:**

The groups share views on the importance of appropriate manpower, careful knowledge management, CDS that fits user workflow, the need for communication among the groups, and for mutual strategizing about the future of CDS. However, views of usability, training, metrics, interoperability, product use, and legal issues differed. Recommendations for improvement include increased collaboration to address legal, manpower, and CDS sharing issues.

**Conclusions:**

The three groups share thinking about many aspects of CDS, but views differ in a number of important respects as well. Until these three groups can reach a mutual understanding of the views of the other stakeholders, and work together, CDS will not reach its potential.

## Background

Because of current U.S. government incentives through the American Reinvestment and Recovery Act (ARRA) [1], hospitals and ambulatory care organizations are increasingly purchasing commercial electronic health record (EHR) systems with computerized clinical decision support (CDS), or they are buying CDS directly from content development vendors. CDS includes “passive and active referential information as well as reminders, alerts, and guidelines [2], p. 524.” Although CDS has been shown to improve healthcare processes and outcomes in a number of studies [3-5], its potential has yet to be reached [6-11]. Challenges to CDS development, management, and use are multi-faceted and complex. They are sociotechnical in nature, involving people, processes, and technology [12]. Key stakeholder groups include healthcare providers and organizations, EHR system vendors, and commercial firms offering CDS content. Prior studies concerning the sociotechnical aspects of CDS have focused on the perspectives of individual healthcare organizations [13-16] or vendors [17] in isolation, with no studies comparing the differing perspectives of each stakeholder group.

Addressing the challenges of CDS development, management, and use requires a system-level view of how multiple perspectives shape the sociotechnical landscape of CDS [17]. To capture a picture of the entire CDS landscape, defined as all activities related to CDS, including views of multiple stakeholders within and outside healthcare organizations, we pose the following research question: How are the views of clinical stakeholders, CDS content vendors, and EHR vendors alike or different with respect to challenges in the development, management, and use of CDS?

## Methods

Because little is known about this topic, an exploratory qualitative research design was selected for answering the research question. Qualitative methods are most appropriate in this case because research has not yet been conducted to identify these challenges posed by CDS. Until the challenges are identified, we cannot know if they are measurable, so measurement at this point is not possible. For example, if more were known about CDS challenges and if they were measurable, a survey of the different stakeholder groups to gain their perspectives might be feasible. However, a valid survey instrument can only be developed if a body of knowledge exists from which questions can be crafted.

We used the Rapid Assessment Process (RAP) as previously described [18] for studying 15 organizations, though we adapted it significantly when studying vendor sites [17]. While ethnography is a mix of methods (usually observation and interviews, but it can include mapping, surveys, and other techniques as well), RAP is a particular type of ethnography which exploits the strengths of using multidisciplinary teams, a combination of quantitative and qualitative data-gathering instruments, engagement of those inside the organization, and strategies for securing high quality data in short periods of time [19]. “Critical elements of the RAP method include: 1) developing a fieldwork guide; 2) carefully selecting observation sites and participants; 3) thoroughly preparing for site visits; 4) partnering with local collaborators; 5) collecting robust data by using multiple researchers and methods; 6) analyzing and reporting data in a collaborative and structured way” [18], p. 299.

### Qualitative research guidelines

This study adheres to the RATS guidelines [20] for qualitative research review, which include principles for relevance of the research question, appropriateness of the qualitative methods selected, transparency of the procedures, and soundness of the interpretive approach.

### Human subjects and the consent process

Institutional review boards (IRBs) at the investigators’ institutions and each clinical site with an IRB approved the study. Specifically, the IRBs representing Oregon Health & Science University, the University of Texas Houston, Kaiser Permanente Northwest, Brigham and Women’s Hospital, HealthEast Care System, LDS Hospital, Providence Portland Medical Center, El Camino Hospital, The Regenstrief Institute (for Wishard), Roudebush VA, and the RWJ Medical Group approved the studies. Caritas and the Mid-Valley IPA ceded authority to Oregon Health & Science University. No patients were included as subjects, so patient consent was not requested. All selected subjects were consented according to requirements of the IRB representing each site. If the site ceded authority to the principal investigator’s site (either OHSU or Brigham and Women’s Hospital), subjects were consented using their approved procedures. If the site required IRB approval in addition, their procedures were also followed. The parent organizations required verbal consent (recorded for interviewees) after the subject was handed a fact sheet about the project and given an opportunity to ask questions about the study. Visited sites sometimes also required written consent using their specified forms, in which case we did both verbal and written consents.

### Step 1 Development of the fieldwork guide

We developed a fieldwork guide, a compilation of tools which includes a site profile form to be completed before a site visit, a schedule of activities to be accomplished before, during and after each site visit, interview guides, forms for use during observational periods with cues for foci, short field survey forms to be completed by 20 to 30 subjects on site through brief interviews to gain a sense of general user perceptions, and agendas for team meetings during site visits. This guide was revised for each site based on what we learned at prior sites, a process reflecting the iterative nature of qualitative studies. Although RAP studies are generally conducted in the field, two of our vendor visits were virtual because subjects were geographically distributed.

### Step 2 Selection of observation sites and participants

For clinical sites, five inpatient and five outpatient sites were selected based on variation in commercial system used, maturity of CDS use, geography, and governance structure [21].

For commercial sites, we purposively [18,22] selected three different types of content vendors so that we could gain a more comprehensive understanding of the issues. Since every hospital we have studied purchases order sets, medication knowledge bases, and clinical information reference resources, we approached three companies that provided those products [17].

To select EHR vendor sites, we asked a group of experts from healthcare and industry who regularly offer advice about our investigations [21] to help select one primarily ambulatory EHR firm and one primarily inpatient EHR vendor with strong CDS products from among those used at one or more of our clinical study sites. This would allow us to directly compare what users and vendors said about the same CDS products.

All study participants were purposively selected. At clinical sites, we selected subjects for interviewing and observing based on their CDS-related roles. We made an effort to seek out clinical champions, normal users, and skeptical users in addition to CDS experts who work to refine, develop, and manage CDS as paid professionals [18]. During content vendor visits, we interviewed individuals in particular roles, including the CEO, vice presidents, content development and management staff, technical/interoperability staff, and informaticians. For the EHR vendors, we targeted staff members who were most involved with CDS.

### Steps 3 and 4 Thoroughly preparing and partnering with local collaborators

In addition to logistical preparation, preparation by the team for each visit included learning about the site’s clinical systems or, in the case of the vendors, the products offered. This was done with the help of an internal collaborator who could assist with any internal human subjects procedures and identify and invite potential subjects. This person also helped arrange for a virtual demonstration of the system or product prior to the site visit.

### Step 5 Collecting robust data by using multiple researchers and methods

Data were collected by a interdisciplinary team of researchers that always included clinicians, social scientists/ethnographers/methods specialists, and informaticians. For each on-site visit, 6–8 researchers, including senior researchers on the team, travelled to a site. Some team members had already worked together for 6 years before starting this study, and others were added over time. They included physicians, nurses, a pharmacist, a laboratory specialist, medical anthropologists, and informaticians with varied backgrounds. Triangulation, or gathering data through multiple lenses from different kinds of subjects, in multiple ways, is a hallmark of qualitative studies. We therefore used a mix of researchers, subjects, sites, and methods for this study.

RAP interviews are semi-structured so there is a definite focus but the interviewee is allowed freedom to elaborate on topics and the interviewer can pursue interesting avenues. They are most often conducted by two interviewers so that one can focus on the interviewee while the other can write fieldnotes, manage the technology, and ask follow-up questions from a different perspective than that of the primary interviewer. All interviews were recorded and transcribed. Observations were conducted in many departments of hospitals, including laboratories and pharmacies, as well as inpatient and outpatient clinical units and exam rooms. Researchers practiced reflexivity [23] by noting personal thoughts in fieldnotes and guarding against leading questions in interviews. Broad areas explored during interviews and observations are listed in Table 1, but a tailored interview guide for each subject was developed prior to each site visit so that questions were relevant for the role of the interviewee. We did not conduct formal observations within vendor organizations to avoid intrusiveness. We visited the three content vendor sites to conduct interviews in person. Interviews of EHR vendor staff members were done by phone because most personnel working with CDS are distributed across the country. We gained further input once we had summarized results by performing member checking [23], whereby we asked representatives of the visited organizations to comment upon our findings.

**Table 1:** **Question topics for CDS study**

**1. Clinical site question areas**	**2. Vendor question areas**
Background and role of interviewee	Background and role of interviewee
The meaning of CDS	The meaning of CDS
Culture and history of organization	Your CDS product or content
Knowledge management practices	About your customers and their use of the product
Governance of CDS	About the marketplace
CDS roles in your organization	CDS roles in your organization
Challenges	Challenges

Data were gathered over a period of five years in three phases. During the first phase, we were funded to study CDS in community hospitals using RAP. Because during that study we found that CDS in ambulatory settings was important yet rarely studied, we sought and received funding to extend our RAP techniques for that purpose. While both of those studies were in progress, we realized that the picture was incomplete without the vendor view. Therefore, we received permission from our funding agencies to use no-cost extensions for site visits and phone interviews with vendor representatives. Over the five-year period, there were a number of changes in the sociotechnical environment of CDS that motivated our continuing interest and our funders’ willingness to support the work. When the first grant proposal was written, few community hospitals and clinics had adopted CPOE with CDS, but later, because of national incentives [1], many more were moving in that direction. Also over the years, the commercial sector increased development and sales of CDS, which encouraged our study of the vendor organizations. The study therefore progressed iteratively as we gained further knowledge and added perspectives.

### Step 6 Analyzing and reporting data in a collaborative and structured way

An inductive thematic content analysis approach was used for analyzing data [18,22,23]. We followed several of the basic tenets of grounded theory in that when were just beginning to explore CDS, we remained very open in our data gathering and analysis. This meant that we conducted line-by-line coding of interview and fieldnote text documents and identified patterns and themes directly from the words of subjects. As we progressed in our analysis over time, we either coded new text into categories we had already established or created new codes, ultimately leading to new or enhanced patterns and themes. All collected data were reviewed during this process. We used qualitative data analysis software (QSR NVivo) with initial coding conducted by pairs of researchers. Each pair consisted of one clinical and one non-clinical researcher. The process included coding one transcript and comparing coding, then discussing and reaching agreement on definitions and meanings of codes before moving on to additional documents. Each pair then presented the final result of its coding to the multidisciplinary team of informatics, ethnographic, and clinical experts. This coding process, as described in a prior paper [18] led to development of the patterns and themes described below. The interpretive process included discussion and eventual agreement about the naming and meaning of themes and subthemes that arose from the data. Using a constant comparative approach, we then analyzed differences across the themes in the clinical, content vendor, and EHR vendor groups to discover the perspective of each group related to each theme.

## Results

### Introduction

We conducted 15 site visits: five to inpatient settings, five to outpatient settings, and five to vendor organizations. We conducted 206 formal interviews with 191 subjects (some were interviewed more than once) and performed 268 hours of observation. We approached approximately twenty subjects who could not consent to an interview because of scheduling conflicts. Table 2 shows the timing of the site visits, type of setting, kind of EHR system, numbers of interviews, number of clinics (for outpatient sites), and hours of observation.

**Table 2:** **Site visit information**

	**Providence Portland Medical Center**	**El Camino Hospital**	**Partners HealthCare**	**Wishard Memorial Hospital Clinics**	**Roudebush Veterans Health Admin.**	**Mid-Valley IPA**	**RWJ Medical Group**	**Zynx Health**	**Eclipsys**	**First Data Bank**	**LDS Hospital**	**UpToDate**	**Caritas Christi Health Care**	**HealthEast Care System**	**NextGen**	**Total**
**Location**	**Portland OR**	**Mountain View CA**	**Boston, MA**	**Indianapolis IN**	**Indianapolis IN**	**Salem OR**	**New Brunswick NJ**	**Los Angeles CA**	**Atlanta, GA**	**South San Francisco CA**	**Salt Lake City UT**	**Waltham, MA**	**Boston, MA**	**Minneapolis/St. Paul MN**	**Horsham, PA**	
**Characteristics of setting**	**Community Hospital**	**Community Hospital**	**Academic and community out-patient**	**Academic and county clinics**	**VA outpatient clinics**	**Community outpatient**	**Academic outpatient**	**Content vendor**	**EHR Vendor**	**Content vendor**	**Community Hospital**	**Content Vendor**	**Community Hospital**	**Community Hospital**	**EHR Vendor**	
**Type of System**	**Commercial**	**Commercial**	**Locally developed and commercial**	**Locally developed**	**Nationally developed**	**Commercial**	**Commercial**	**NA**	**NA**	**NA**	**Locally developed**	**NA**	**Commercial**	**Commercial**	**NA**	
**Date of Visit**	**12/2007**	**2/2008**	**6/2008**	**9/2008**	**9/2008**	**12/2008**	**2/2009**	**7/2009**	**9/2009**	**10/2009**	**1/2010**	**3/2010**	**3/2010**	**7/2010**	**5/2011**	
**Hours observing**	**36**	**26**	**37**	**20**	**25**	**33**	**26**	**NA**	**NA**	**NA**	**19**	**NA**	**17**	**29**	**NA**	**268**
**Individuals observed**	**10**	**12**	**17**	**16**	**17**	**27**	**17**	**NA**	**NA**	**NA**	**15**	**NA**	**12**	**12**	**NA**	**155**
**Number of clinics observed**	**NA**	**NA**	**9**	**6**	**5**	**9**	**6**	**NA**	**NA**	**NA**	**NA**	**NA**	**NA**	**NA**	**NA**	**35**
**Number of interviews**	**15**	**12**	**13**	**9**	**9**	**9**	**12**	**6**	**10**	**6**	**18**	**9**	**25**	**41**	**12**	**206**

Table 3 shows the roles of subjects. We have attempted to categorize them, but since roles are overlapping, titles are varied, and backgrounds are extremely diverse [24], the assignment to categories can only be based on our best judgment. We define the categories as follows. MD informatics personnel work primarily in informatics, including in content development, though some also practice medicine part time. Other clinical informatics staff members are also primarily informaticians but are nurses, pharmacists, and others with clinical backgrounds. Information technology and non-clinical informatics employees include IT staff and informaticians without clinical degrees. Clinicians with some informatics responsibilities are physicians, nurses, pharmacists, or others who spend time in informatics roles either formal or informal, including chairing or serving on CDS committees. Health information management (HIM) and administrative roles are those held by certified HIM professionals, quality improvement leadership, and administrators not at the vice presidential level. CEO/VP/CMO denotes chief executive officer, vice-president, or chief medical officer, all higher-level leadership positions, including those in the commercial organizations. Finally, the content development and business development/marketing designations are restricted here to the staff in vendor organizations.

**Table 3:** **Roles of subjects at each site**

	**Health East Care System**	**Caritas Christi Health Care**	**LDS Hospital**	**Providence Portland Medical Center**	**El Camino Hospital**	**Partners Health-Care**	**Wishard Memorial Hospital Clinics**	**Roudebush Veterans Health Admin.**	**Mid-Valley IPA**	**RWJ Medical Group**	**Zynx Health**	**First Data Bank**	**UpToDate**	**Eclipsys**	**NextGen**	**Total**
**MD informatics**	**2**		**1**	**1**	**1**	**6**	**7**	**1**	**1**	**1**						**21**
**Other clin informatics**	**24**	**3**	**1**	**2**	**5**	**1**	**1**	**3**		**1**						**41**
**IT and non clin informatics**	**10**	**7**	**1**		**4**	**1**	**1**	**3**	**5**	**5**						**37**
**Clinical, some with informatics**		**7**	**8**	**3**	**3**	**5**		**1**	**2**	**2**						**31**
**HIM, admin**	**2**	**1**			**2**					**2**						**7**
**Clin admin, some in quality**	**3**	**2**		**1**	**3**			**1**	**1**	**1**						**12**
**CIO/IT**		**1**		**1**									**2**	**1**	**3**	**8**
**CEO/VP/CMO**		**2**	**2**	**2**							**2**	**3**	**4**		**1**	**16**
**Content development**											**2**	**1**	**2**	**7**	**1**	**13**
**Total**	**41**	**23**	**13**	**10**	**18**	**13**	**9**	**9**	**9**	**12**	**4**	**4**	**8**	**8**	**5**	**186**

**Figure 1:**
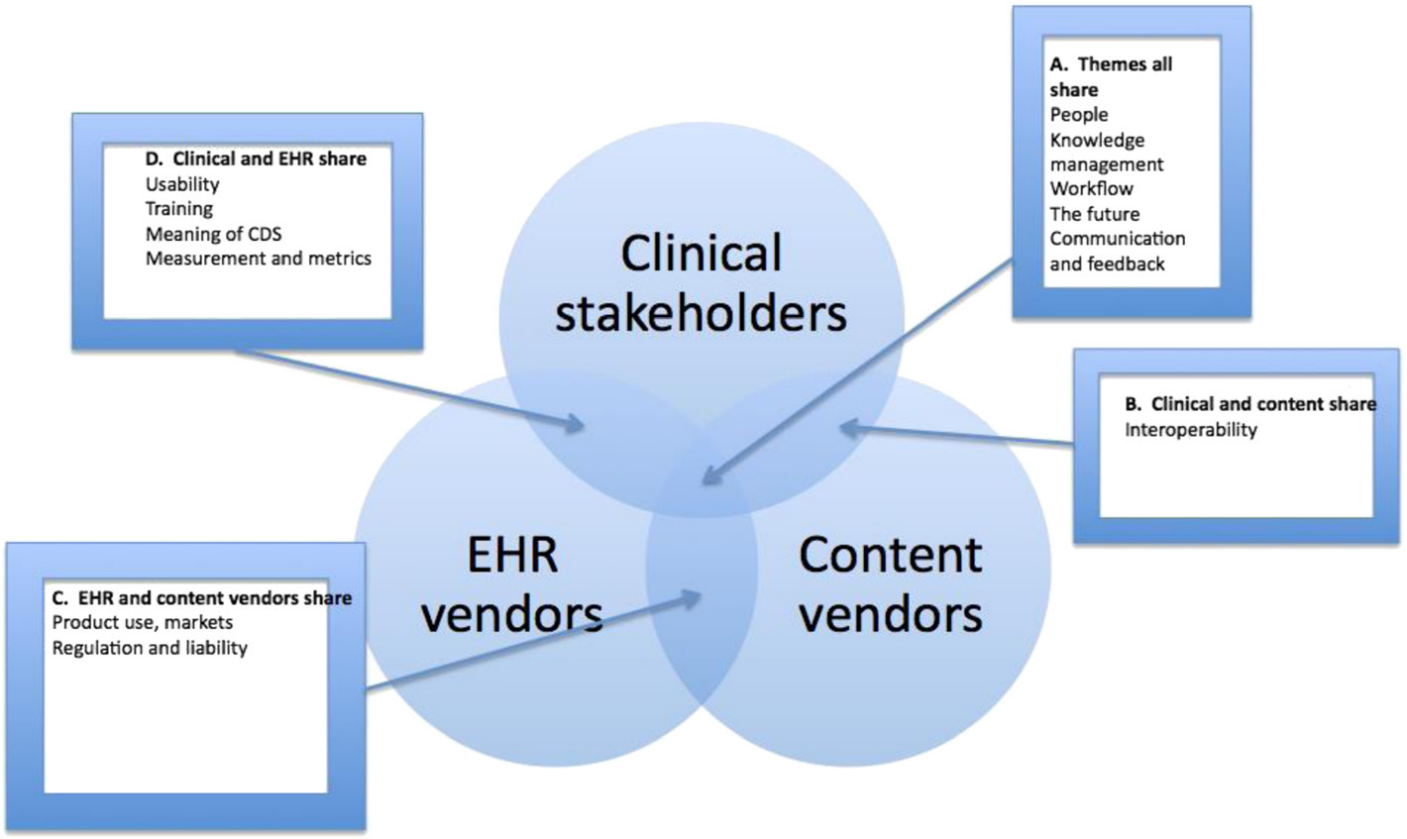
Themes shared by three groups.

Figure 1 outlines the major themes that were shared by the three groups. We have described themes from the clinical perspective [21] and the content vendor perspective [17] in prior publications. Here, we will limit our descriptions to themes shared by two or more of the stakeholder groups.

In the following description of results, the quotes, which illustrate patterns, are representative of what we heard from numerous sources. Brackets are placed around our explanations to improve readability or where we removed proprietary terms.

## Themes shared by all three groups

Please see box A in Figure 1.

### Theme 1: People

All three groups told us about new kinds of essential people needed for CDS [24]. These include knowledge engineers, analysts who modify content at clinical sites, clinicians specializing in evidence-based medicine at content vendor sites, and usability and interoperability experts at content vendor sites. The clinical organizations need analysts with both technical skills and knowledge of healthcare to customize CDS. Content vendors need clinicians with skills in evidence-based medicine and writing; EHR vendors need physician consultants who help train staff within purchasing organizations to manage CDS and use it. The notion of embedded employees and the need for vendor staff members to understand healthcare issues are two special points of interest at the intersection of the three “workforces”.

#### Embedded employees

Many healthcare sites have at least one employee on site paid by the EHR vendor or who formerly worked for the vendor. One hospital site has numerous employees who formerly worked for the EHR vendor and now work for the hospital, while others (one a hospital and one a group of clinics) have outsourced nearly all IT and informatics responsibilities to their vendors. The allegiances of embedded employees are sometimes more towards either the clinical site or to the vendor; these staff members often feel conflicted.

#### Vendor employees’ understanding of healthcare

Clinical customers frequently criticized EHR vendor employees for not having a comprehensive enough understanding of healthcare to be able to develop or productively customize an EHR or reports. CDS analysts at clinical sites are therefore especially delighted when they can deal with vendor staff members who have clinical backgrounds. Physician consultants working for EHR vendors are highly valued by physician customers. Those representing healthcare organizations felt strongly that vendors should hire more staff members with clinical backgrounds.

### Theme 2: Knowledge management

For the clinical sites, knowledge management includes not only selecting and modifying CDS purchased from vendors, but also developing CDS, inventorying, updating, and continuously monitoring usefulness of CDS. For CDS content vendors, knowledge management is essential for their ability to produce and maintain content. Content vendors all have proprietary mechanisms for keeping track of CDS within their organizations. They also provide software that clinical sites can purchase to help them to manage CDS. There are three aspects of knowledge management (listed below) that illustrate tensions among clinical sites, content vendors, and EHR vendors.

#### Customization of CDS

Clinical site staff members are often frustrated that they have to put so much effort into even the most basic CDS. Modifying CDS so that it suits the local work process is time-consuming. A content vendor representative noted: “I don’t care if it’s a community [hospital] or large [hospital], they don’t have the resources or the bandwidth to do this [CDS]”.

#### Content vendor products

Most hospital representatives purchase some content directly from content vendors and are often frustrated with the products. They are concerned that content contains too many options, is not current enough, or is not good enough (i.e., evidence-based).

#### Sharing content

Representatives of all groups thought that sharing of CDS would be an ideal but hard to reach goal. Clinical sites rarely share CDS content they have developed. A hospital CDS analyst said: “Some things are hard to share. So you just get like a bunch of screen prints. . . somebody has to actually go and program that or configure that, and it may be a six-month effort.” Some vendors enable sharing by providing web sites and discussion mechanisms for this purpose. Even so, some clinical sites do not freely share because they would like compensation or they believe sharing could cause liability problems. Another hospital analyst noted that an EHR vendor may be averse to the idea of customers sharing content they paid for but the vendor developed for them: “[CDS rules] created by the vendor consultant are the client’s property but should not be shared because the vendor wants to continue to make money off it [i.e., by selling the content to other healthcare customers]”.

### Theme 3: Workflow

All three groups are cognizant of the impact of CDS on the workflow of end-users, although their views differ slightly. The clinical organizations are especially sensitive about providing CDS that will frustrate physicians by slowing their work with an inordinate number of alerts or reminders. The vendors are all striving to improve workflow by providing more active decision support that is embedded within the EHR so that the clinician does not have to ask for it separately.

### Theme 4: Communication and feedback

In this section, we focus on communication and feedback among the three groups.

#### Collaboration

Collaborative communication between clinical site employees and vendor employees ranges from daily to infrequently. Some have good, close working relationships, while others experience friction. Clinical staff members who manage to find individual vendor staff members to call on a consistent basis believe that is key.

#### Open dialogue

All three content vendors point to the need for open dialogue between content vendors, EHR vendors and end users. However, dialogue is time-consuming and difficult, and because the EHR vendors are often customers of content vendors, it can be especially complicated for them to speak freely. An EHR vendor representative praised the work of the HL7 standards body for promoting open dialogue in a legally safe environment: “in HL7 meetings you would have the best developers from each company come together and they would share ideas in a non-competitive environment”.

#### Misunderstandings

All three groups believe the other groups do not understand what they do. Clinical sites do not understand content vendors because they do not know how much effort goes into developing CDS content; content vendors do not understand the burden on the clinical sites to modify the content; EHR vendors do not understand how difficult it is for content vendors to integrate their products into EHRs that follow different interoperability standards.

#### Vendor neutrality

Content vendors discussed the business necessity of staying (and appearing) neutral toward both EHR vendors and end-users, given that EHR vendors are also (competing) customers. They cannot afford to alienate potential or actual customers.

#### User feedback

Some vendors of both types have difficulty gaining useful feedback from end-users or simply do not try. Several strive to gather informal feedback from customers. One actively solicits feedback through formal customer satisfaction surveys and random spot-checking during implementation. Interviewees representing this vendor also talked about involving stakeholders in developing requirements for designing and developing CDS. They also monitor customer use of the product to make decisions regarding future content.

### Theme 5: The future, vision, and strategy

All three groups share optimism about the future.

#### Corporate strategy

The companies want to continue to grow and change to keep a competitive edge. They are working to identify new markets and strategies and to pursue them aggressively. They are offering varied packages to meet diverse customer needs. These include different kinds of licensing, training, and hosting services. They aim to be prepared for and able to take advantage of changes in federal policy. Interviewees from both EHR companies expressed the idea that having a robust data analytics capacity was a main selling point for their respective companies.

#### Perceptions of the other

Content vendor representatives felt that because of Meaningful Use there is an emerging understanding of “what [they] do” on behalf of both the public and customers, and that this would lead to greater customer appreciation. The vendor representatives’ view of customers is mainly positive in that they admire the hospitals and clinics for being at the forefront of EHR implementation. However, there are some undercurrents of disappointment as well. One vendor representative expressed frustration that the customers were motivated more to increase their revenue than to improve patient care: “So while we have decision support . . . the bulk of it supports making sure they get paid.” Vendors told us customers are not clamoring to purchase CDS that focuses only on improving the quality or safety of care. Customers, they believe, often expect the software to do more than it can, but use of these products is often suboptimal. A content vendor employee said: “And I’ve seen several times where the client is trying to make the products work differently… to meet their ideal workflow in their mind and they’re fighting the product”.

## Themes shared by clinical and content vendor groups

Please see box B in Figure 1.

### Theme 6: Interoperability

Both clinical site and content vendor groups are frustrated that more progress has not been made by EHR vendors regarding interoperability and use of standard protocols. With respect to CDS, the content vendors would like it to be easier for their products to be embedded within different commercial EHRs: “It can be sometimes a little frustrating when their format is just different than ours.” There is a history of content vendors being bought by EMR vendors and later becoming separate again [25-28], so in these cases there remains some interoperability. One content vendor representative recommended: “The optimal way for clinical content and EHR vendors to work together is to keep separate, but standardize the interfaces between them”.

#### Interoperability and the relationship of clinical sites with content vendors

Customers resent the large amount of work and resources necessary to fit specific content into an EHR. Because of this demand on resources, EHR vendors tend to have relationships with specific content vendors. Although it may be theoretically feasible for a customer to use a different content vendor than one that is partnered with their EHR vendor, it is often both more difficult and costly.

#### Interoperability and the relationship of EHR vendors and content vendors

Interoperability between EHR and content vendor products was described by interviewees as necessary but challenging. A content vendor representative noted the need for collaborating: “But at times you really do need to come together to say, you know, we are doing this together for a site”.

## Themes shared by EHR and content vendor groups

Please see box C in Figure 1. These subthemes include market considerations and also regulations and liability concerns.

### Theme 7: Markets, products and customers

Because a number of our interviewees at vendor sites were in marketing, we were able to discover their views on market segmentation, what products appeal to certain markets, and how they view their customers. Subthemes include: who is the customer, integration, and competition.

#### Who is the customer

All vendors talked about how they define “the customer,” the varied and complex relationships they have with these customers, and the differences in expectations and approach required by each. Customers include EHR vendors that are customers of a content vendor. Health systems, hospital IT departments, and end-user clinicians may all be customers of either content vendors or EHR vendors.. To make matters even more complicated, agreements and types of relationships often vary even within a single customer category.

#### Integration

The product that content vendors produce must be able to be integrated into multiple EHRs in order to be commercially successful. Integrating content has implications for product design and underscores the need for effective relationships with EHR vendors.

#### Competition

The overall vendor environment is increasingly competitive and deliberately non-integrated. In order to remain competitive as a business, the vendor must clearly brand products and keep pace with changing market expectations. The primary niche identified by content vendors is providing evidence-based content. Content vendors also felt that most people did not fully understand what they do, and how resource-intensive the work is.

#### Liability for content

The EHR vendors and the content vendors clearly state that they do not practice medicine and therefore should not be liable for decisions that can only be made by clinicians. They provide a vehicle for using CDS content, but it is the responsibility of the clinician to decide the medical relevance of that content for the patient’s care.

## Themes shared by EHR vendor and clinical stakeholder groups

Please see box D in Figure 1.

### Theme 8: Usability

We heard constant and strongly worded complaints from the clinical site representatives about the usability of all vendor-developed EHRs. We observed continuous usability problems, defined as difficulties experienced by the user directly interacting with the clinical information system, which can impact the effectiveness of CDS. The EHR vendor representatives noted that their organizations were paying a great deal more attention to usability than ever before. One of them said: “Well, you know, I mean certainly in the past four or five years the user interface, user experience type people have become more in demand by vendors and that’s probably going to continue to grow as we hear more noise about usability”.

### Theme 9: Training

Interviewees described 1) how training happens, 2) training options (mostly provided by vendors), 3) training for different types of clients (e.g. virtual training for small clients), 4) the role and purpose of training, and 5) the people who do the training. At the clinical sites, vendors often do training. In the case of large clinical organizations, the EHR vendors train trainers within the organization. They may also train analysts and IT staff within those organizations. In smaller organizations, the vendors may train users directly. The clinical site employees had mixed feelings about the quality of training they received from vendors. Conversely, the vendor representatives complained that often, clinical organizations were too reluctant to pay for training.

### Theme 10: The meaning of CDS

Within the clinical sites, we found that the term CDS meant different things to different study subjects [29]. Many clinical users were not familiar with the term at all. Those who did know the term described it in either very narrow terms (e.g. alerts and reminders) or broad terms such as guidance provided by the EHR for making clinical decisions. Because our study subjects within the vendor organizations were selected due to their knowledge of CDS, they offered careful and detailed descriptions of what CDS means to them. They universally believe a broad definition that includes population-based reports and analytics in the definition.

### Theme 11: Measurement and metrics

Measurement of the use and effectiveness of CDS is a challenge. Representatives at one clinical site discussed how they had difficulty accessing data for reporting because the data were maintained in a proprietary format by their vendor. Other representatives perceived that the difficulty in their ability to generate reports was related to their vendor’s preference to be paid to generate reports for them.

One vendor has offered free analytics capabilities for a number of years, yet few customer organizations have used them. However, interest among customers is increasing rapidly. An EHR vendor representative said: “many practices are interested in quality measures because of reimbursement, but many are also interested in improving their care”.

## Discussion

The three major stakeholder groups involved in CDS share views on the importance of appropriate manpower, careful knowledge management, CDS that fits user workflow, the need for communication among the groups, and for mutual strategizing about the future of CDS. However, views of usability, training, metrics, interoperability, product use, and legal issues differed.

### Strengths and weaknesses of this study

The major strengths of this study include the breadth and variety of 1) organizations selected as sites, and 2) subjects selected for observation and/or interviewing. This study is unique in involving commercial as well as health care entities as sites. The breadth of backgrounds represented on the research team and the team’s experience using RAP also strengthened the study. Other strengths, including the ability to include senior investigators in all six RAP steps for all site visits over five years, and to conduct especially rigorous research, were made possible by U.S. government funding.

Limitations include the length of the study. It is possible that, because the 15 visits spanned a period of five years, some of the earlier subjects may now have different opinions than what was shared in their interviews, particularly since ARRA incentives began during that period. On the other hand, we carefully considered the temporal factors during analysis, and this enhanced our understanding of the fundamental, enduring, broad themes that were generated. Another limitation of this study was only being able to conduct telephone interviews at some vendor sites. In person interviews are always preferred, but subjects belonging to these organizations were not co-located. Although we included three major stakeholder groups in our landscape, we were unable to gather data directly from what may be the most influential factor, that of the government regulators who created the legal environment surrounding these groups. Finally, because this was a qualitative study, the results are not generalizable to all clinical and vendor organizations, but they should be transferable to entities similar to those studied.

### Implications of results

There are many tensions among the three groups that make up the landscape of CDS. Some tensions relate to misunderstandings among the groups. However, subjects told us that the most severe tensions stem from the external characteristics, such as the need for national standards for interoperability that are actually used by vendors. Standards would provide a safe, legal environment in which vendors would be empowered to provide more CDS tools to customers. There is progress being made by standards groups and our results underscore the importance of this work.

Interviewees want more collaboration and open dialogue. The CDS content vendors we studied believe that they are neutral developers of clinical content which EHR vendors and clinicians consume. They would like to have more interaction with the other players in the CDS landscape. To promote adequate understanding among the most important entities critical to successful use of CDS, a “three-way conversation” among content vendors, electronic health record vendors, and users was recommended by a number of interviewees. These conversations would help to achieve the mutual goal of better healthcare assisted by CDS. Standards groups provide an appropriate venue for such conversations, as do professional associations, especially the American Medical Informatics Association (AMIA) and HIMSS.

### Relationship to other studies

Many interviewees also outlined legal issues that impede sharing and collaboration. Authors of prior publications have promoted this idea [30,31] and there has been some progress. For example, the Office of the National Coordinator for Health Information Technology has commissioned a research group to identify a small sub-set of medication interaction rules that are especially useful [32]. Such pre-selection could help users to prioritize CDS knowing there is general agreement about some high-impact alerts. Since this would represent a standard of care, there could be some legal protection at least for the use of these alerts.

Most subjects outlined advantages of increased sharing. Currently, there is no easy way for healthcare organizations to share the clinical content. Clinical organizations cannot continue to develop and redevelop the same CDS interventions. Many interviewees felt there should be a mechanism that allows organizations to share content and provides legal protections to the organizations. Prior authors have outlined how this has been accomplished in pilot studies [33-37].

The health care organizations plan to measure CDS effectiveness increasingly in the future, and to improve their monitoring and reporting. The vendor organizations are developing additional tools to help them. The publication of the six systematic reviews by Haynes et al. [6-11] made it clear that all parties involved in the design, development, implementation, and use of CDS interventions need to develop metrics if CDS is to be evaluated in a rigorous fashion (e.g., RCTs, usage statistics) [38].

### Future research

A follow-up study after the impact of ARRA [1] becomes clearer could offer an assessment of how issues identified in the present study are being addressed and what challenges remain. It would be especially useful if the study could discover the perspectives of both public and private regulatory and accrediting bodies concerning CDS, in addition to the clinical and commercial stakeholder groups included here.

## Conclusions

By conducting site visits to 15 organizations representing clinical users, content vendors, and EHR vendors, we gathered data on the broad landscape that impacts challenges to CDS development, management, and use. The three groups share thinking about many aspects of CDS, but views differ in a number of important respects as well. Until these three groups can reach a mutual understanding of the views of the other stakeholders, and work together, CDS will not reach its potential.
